# Medical Items

**Published:** 1915-12

**Authors:** 


### MEDICAL ITEMS

W. B. Saunders Company, Publishers of Philadelphia and London, have just issued their 1916 eighty-four page illustrated catalogue. As great care has evidently been taken in its production as in the manufacture of their books. It is a descriptive catalogue in the truest sense, telling you just what you will find in their books and showing you by specimen cuts, the type of illustrations used. It is really an index to modern medical literature, describing some 300 titles, including 45 new books and ned editions not in former issues.

A postal sent to W. B. Saunders Company, Philadelphia, will bing you a copy—and you should have one.

---

A Chicago physician reports:—

"Some time since a young lady patient of mine learning that her mother, who lives in New York, was so very ill from neuralgia and general prostration that the case was considered alarming, as no relief could be obtained except from the use of narcotics, asked my advice and I told her that while I was not at liberty to interfere, if her doctor was willing, she should try Tongaline. I have just learned that the mother did so and is now so much improved as to be able to go to the Adirondack Mountains to recuperate."

### GONORRHOEAL URETEROPYELITIS—ANTIMENINGITIC SERUM.

In "Surgery, Gynecology and Obstetrics" for October, Dr. Montague L. Boyd of Atlanta discusses the treatment of complications of Gonorrhoea by Antimeningitic Serum and reports the results of two cases of Ureteropyelitis in which the treatment was of avail. Although the method has been used there has been but one case of this particular complication re-there has been btu one case of this particular complication reported. The satisfactory results obtained warrant the mention of two more cases.

The doses were given subcutaneously and varied from ten to twenty ccm; the total amounts were 42 ccm in one case and 21 ccm in the other.

The clinical history and the progress of the treatments are reported in detail and mark a step forward toward the relief of a burdensome complication.

---

### GALL STONES AND ROENTGEN RAY.

In "Some Remarks on the Detection and Diagnosis of Gall Stones by the Roentgen Ray," by Dr. George M. Niles of Atlanta, appearing in the Southern Medical Journal for December, the writer shows that the exploration of the gall bladder by the Roentgen Ray is of decided value when carefully done.

The technique of the work must be careful and exact and the interpretation of the plates must be painstaking and exhaustive. Many plates have to be made. In one case the 18th plate out of 21 showed a singe stone and the stone was found later by the surgeon. This is expensive, but it has to be done until experience and science show a quicker way. As it is, the results warrant the labor and the guide the surgeon as well as the internist has from the plates is invaluable. The study of gall stones in this manner is still in the pioneer stage and these steps are of the utmost importance.

In a majority of chronic dyspepsias this exploration by the Roentgen Ray is worth while.

